# Editorial: Advances in theranostics: Novel nanotools for the treatment and diagnosis of tumors

**DOI:** 10.3389/fbioe.2023.1143857

**Published:** 2023-02-03

**Authors:** Roberta Censi, Piera Di Martino

**Affiliations:** ^1^ School of Pharmacy, Drug Delivery Division, University of Camerino, CHiP Research Center, Camerino, Italy; ^2^ Department of Pharmacy, University of Chieti, Chieti, Italy

**Keywords:** nanoparticles, oncology, smart theranostics, breast cancer, new diagnostic agents, stimuli responsive biomaterials

The Research Topic entitled “*Advances in Theranostics: Novel Nanotools for the Treatment and Diagnosis of Tumors*” presents a small series of articles reporting the most exciting research, novel application studies and scientific progress in the field of nanostructures and functional materials used in the field of oncological theranostics. The limitation in the effectiveness of traditional approaches in treating cancer are largely demonstrated in this Topic Research Topic, providing examples of stimuli-sensitive and technologically advanced novel nanoplatforms (nanoparticles, liposomes, micelles, polymer-drug conjugates, and dendrimers) that, in a multidisciplinary and collaborative approach, involve the contribution of different disciplines ranging from material sciences, biology, immunology, medicine as well as diagnosis. Nanotechnology platforms that can combine both therapy and diagnosis are especially interesting, as this approach allows combining targeted therapy and monitoring of efficacy with minimal invasiveness and systemic toxicity. Multifunctional nanomedicines combining responsiveness to tumor microenvironment, targeting, therapy, sensitivity, and early-stage detection are highly advantageous and are most likely the future clinical direction of cancer management. The high number of views obtained by the articles published in this Research Topic Collection shows a clear interest of the scientific community for nanomedicine and theranostics and motivated the edition of this Topic Research Topic. Our goals are to facilitate access to thematically related papers and draw attention to the current activity and trends in addressing remaining challenges, ultimately serving as a valuable resource to the biomedical and pharmaceutical science community and beyond. The Research Topic is comprised of four selected peer-reviewed manuscripts (2 reviews and 2 original research articles) derived from the fields of (bio) materials science and engineering, pharmaceutics, and biology.

The review paper by Cong et al. opens this Topic Research Topic by overviewing the abnormal pathophysiological characteristics of the tumor microenvironment, including acidosis, overexpression of special enzymes, hypoxia, and high levels of ROS, GSH, and ATP. These characteristics are exploited in drug delivery systems (DDSs) design to trigger the spatial and temporal control of drugs and/or diagnostic agents in tumor tissues. The latest advances in the field of DDSs responsive to stimuli found in tumor microenvironment are highlighted in this review paper, emphasizing their ability to effectively realize tumor-site specific drug, decrease the injected dose and systemic toxicity. Finally, a critical analysis of the existing bottlenecks to the clinical translation of these technologies are also critically discussed and include, among others, DDS complexity, sub-optimal biocompatibility, patient biological heterogeneity, leading to varying response, size control and immune system reaction.

An example of stimuli-sensitive nanoDDS for tumor diagnosis and treatment if offered by Xu et al., who proposed a nanoparticle-based platform for the effective presentation of sonosensitizers to hypoxic tumor tissues. In this work, a core-shell structured nanoparticle (IR780/PLGA@MnO2NPs) loaded with IR780 and manganese dioxide (MnO2) was developed as a nanocarrier to transport the sonosensitizer IR780 and the generated oxygen into the tumor tissue. The MnO2 shell layer of IR780/PLGA@MnO2NPs was designed to respond to tumor microenvironment, by releasing IR780 and generating reactive oxygen species in response to tumor acidity and excess of H2O2. As a result, the generated oxygen relieves tumor tissue hypoxia and kills tumor cells, while the generated Mn enhances magnetic resonance imaging (MRI) signal intensity by acting as a contrast agent for MRI. The developed multi-layer core-shell nanostructure was validated *in vitro* and *in vivo* on a breast cancer mice model, providing a promising strategy for cancer diagnosis and sonodynamic treatment, with potential future application in cancer theranostics.

Ranjbari et al. described nanostructure two-dimensional Mxene as potential materials in the diagnosis and treatment of breast cancer. Different synthetic methods for producing biocompatible Mxenes and their application to the detection and therapy of breast cancer are overviewed. The authors point out the high biocompatibility profile of. MXenes’ and their surface modification flexibility, leading to multifunctional properties, such as preferential agglomeration at tumor sites for photothermal treatment by the non-covalent reactions on the MXene surface with PEG, CS, SP, and PVP materials. The synthetic MXenes could be potentially applied in the fields of, antimicrobial materials, attributes, drug delivery, engineering of tissue, and extensive near-infrared sorption.

Breast cancer is also tackled in the original research article by Wang et al., dealing with the assessment of the diagnostic value of 18F-NaF PET/CT in diagnosing bone metastases in patients with nasopharyngeal carcinoma (NPC) using, for the first time, visual and quantitative analyses. The study was carried out retrospectively, analyzing 164 patients with NPC who underwent 18F-NaF PET/CT between 2017 and 2021. They showed that visual analysis of 18F-NaF PET/CT findings is accompanied by high sensitivity and specificity for the diagnosis of bone metastases in NPC. Furthermore, the quantitative analysis of PET/CT (SUVmax), displaying the advantage of higher objectivity and reproducibility than visual qualitative analysis, was demonstrated a valuable tool for the differential diagnosis between bone metastases in NPC and benign bone lesions.

In conclusion, this Research Topic Collection represents a small cross-section of the research work performed around the topic of novel nanotools for the treatment and diagnosis of tumors, having the scope to promote ideas, show new technologies, present the state of the art in the field, and critically discuss current challenges still existing in this area and possibilities for overcoming these challenges. We are convinced that this article Research Topic will be significantly relevant to the scientific community and beyond to spotlight cutting edge studies and start initiatives and collaborations among scientists in this field.

This Research Topic aims at collecting recent advances in theranostic nanoplatforms, with a special emphasis on originally designed systems. Nanoplatforms combining drug delivery, targeting, and tumor monitoring approaches could be of particular interest when designed to address the challenge to specifically reach tumors in specific body districts, or treat tumors of different stages.

